# Minimally invasive vs. open hepatectomy in patients with obesity and liver tumors: a systematic review and meta-analysis

**DOI:** 10.3389/fsurg.2026.1718991

**Published:** 2026-04-15

**Authors:** Song Jiang, Wanjuan Li, Hongwei He, Yongchuan Huang, Wensong Liu, Qi Zheng

**Affiliations:** Department of Hepatobiliary Surgery, Jinshan Branch of Shanghai Sixth People’s Hospital, Shanghai, China

**Keywords:** liver tumor, meta-analysis, minimally invasive hepatectomy, obesity, open hepatectomy

## Abstract

**Background:**

Minimally invasive hepatectomy (MIH) has been considered a safe and feasible treatment approach for liver resection in recent years. However, the application of MIH in patients with obesity still remains controversial, and the outcomes of MIH vs. open hepatectomy (OH) have not been fully evaluated. Our objective is to compare the surgical outcomes of MIH with OH in patients with obesity and liver tumors.

**Methods:**

All studies comparing MIH with OH in patients with obesity and liver tumors were identified through a systematic search of the PubMed, Embase, Web of Science, Cochrane Library, WanFang, and CNKI databases. Statistical analysis was conducted using Review Manager version 5.4 software. The final search was conducted on 20 May 2025. The surgical outcomes included operative time, blood loss, blood transfusion rate, tumor size, overall and major postoperative complications, biliary leakage, liver failure, mortality, and length of hospital stay.

**Results:**

Eleven studies with a total of 1,713 patients were included in this meta-analysis. Compared with OH, MIH was associated with a shorter operative time [weighted mean difference (WMD) = −54.50, 95% confidence interval (CI) = −96.28 to −12.72, *P* = 0.01], reduced blood loss (WMD = −416.80, 95% CI = −579.84 to −253.76, *P* < 0.00001), and a lower blood transfusion rate (OR =  0.31, 95% CI = 0.19–0.48, *P* < 0.00001). In addition, MIH was associated with lower rates of overall complications (OR =  0.60, 95% CI = 0.48–0.75, *P* < 0.0001), major complications (OR =  0.61, 95% CI = 0.42–0.89, *P* = 0.01), biliary leakage (OR =  0.48, 95% CI = 0.26–0.88, *P* = 0.02), and liver failure (OR =  0.26, 95% CI = 0.08–0.93, *P* = 0.04) and shorter postoperative hospital stay (WMD = −7.21, 95% CI = −10.22 to −4.21, *P* < 0.00001). Tumor size was smaller in the MIH group (WMD = −1.06, 95% CI = −1.70 to −0.41, *P* = 0.001). However, there was no significant difference in mortality between the MIH and OH groups (OR =  0.68, 95% CI = 0.45–1.03, *P* = 0.07).

**Conclusions:**

The results suggested that MIH is associated with shorter operative time, reduced blood loss, lower blood transfusion rate, decreased rates of overall complications, major complications, biliary leakage, and liver failure, and a shorter hospital stay. However, no significant difference in mortality was observed between the MIH and OH groups. MIH appears to be a safe and effective treatment option for patients with obesity and liver tumors.

**Systematic Review Registration:**

https://www.crd.york.ac.uk/prospero/display_record.php?ID=CRD42024502015, PROSPERO CRD 42024502015.

## Introduction

The prevalence of overweight and obesity is increasing worldwide (1). The number of individuals with obesity more than doubled between 1990 and 2022. In 2022, over 890 million adults were living with obesity (2). Meanwhile, the burden of obesity-related disease continues to rise (3). Studies have suggested that a high body mass index (BMI) ranks as the sixth most common risk factor contributing to the global burden of disease (4) and is among the top five risk factors for attributable deaths worldwide (3). Compared with individuals of healthy weight, people with obesity experience a reduction in disease-free life of 3–8 years and have an approximately 1.3-fold higher risk of premature death (5). In addition, obesity predisposes people to a series of clinical conditions, such as type 2 diabetes, cardiovascular disease, chronic kidney disease, and several types of cancers (6, 7).

Studies have confirmed that obesity, characterized by adipose tissue accumulation, is associated with low-grade systemic inflammation (8, 9). Hypertrophic adipocytes and tissue-resident immune cells undergo phenotypic changes, ceasing the secretion of anti-inflammatory and protective cytokines and beginning the secretion of inflammatory adipokines and cytokines that exert both local and systemic effects (9). Despite its low-grade feature, adipose tissue inflammation can negatively impact the function of distant organs and is considered a cause of obesity-related complications (9, 10). Furthermore, studies have shown that in patients with obesity, the degree of obesity is independently associated with increased postoperative complication rates compared with patients without obesity (11–13). Moreover, adipose tissue inflammation and obesity-associated alterations in the tissue microenvironment are considered to exert tumor-promoting effects (14).

Minimally invasive hepatectomy (MIH), including laparoscopic and robotic approaches, has advanced significantly in liver surgery in recent years. Owing to the magnified visualization provided by minimally invasive techniques, and particularly the stability, tremor filtration, and enhanced instrument flexibility of robotic platforms, MIH allows for gentle manipulation of the liver and precise tissue dissection (15, 16). A study demonstrated that patients undergoing MIH experience less blood loss, lower morbidity, and a shorter length of hospital stay than those undergoing open hepatectomy (OH) (17).

However, the outcomes may differ in patients with obesity when hepatectomy is conducted using minimally invasive approaches. First, obesity increases the difficulty of liver resection and the risk of conversion due to inadequate exposure of the surgical field (18). Second, these patients may at increased risk of intraoperative complications related to the use of CO_2_ pneumoperitoneum. For example, elevated intra-abdominal pressure can enhance venous stasis, reduce respiratory compliance, increase airway pressure, and impair cardiac function (19). Furthermore, the prognosis of MIH in patients with obesity remains controversial (20, 21).

Currently, to our knowledge, no meta-analysis has been conducted to compare the surgical outcomes of MIH and OH in patients with obesity and liver tumors. Considering these aspects, we performed this study to evaluate the safety and effectiveness of MIH in this patient population.

## Materials and methods

The work was conducted in accordance with the PRISMA (Preferred Reporting Items for Systematic Reviews and Meta-Analyses) guidelines (22) and was registered with PROSPERO (registration number: CRD42024502015).

A literature search was conducted in the following databases: PubMed, Embase, Web of Science, Cochrane Library, WanFang, and CNKI. The search terms included the following: [laparoscopic OR laparoscopy OR robotic OR robot OR da vinci OR minimally invasive OR minimal invasive surgery] AND [open OR traditional open surgery] AND [hepatectomy OR liver resection OR liver surgery OR hepatic surgery] AND [obesity OR obese patients OR adiposity OR body mass index (BMI)]. The reference lists of the selected studies and relevant systematic reviews were also screened to identify additional studies that met our inclusion criteria. The final literature search was performed on 20 May 2025.

## Inclusion and exclusion criteria

### Inclusion criteria

The inclusion criteria for this meta-analysis were as follows: (1) study comparing MIH with OH in patients with obesity and liver tumors; (2) studies reporting at least one of the outcomes of interest; and (3) studies in which each group included at least 10 patients.

### Exclusion criteria

The exclusion criteria were as follows: (1) studies without a control group; (2) non-human studies, reviews, letters, abstracts, editorials, and case reports; and (3) studies comparing patients with obesity to those without obesity.

### Data extraction and outcomes of interest

Two authors screened the titles and abstracts of the studies identified through the search strategy. The full texts of potentially eligible studies were retrieved and assessed according to the inclusion criteria. The final selection of included studies was determined by consensus between the two authors. In cases of any discrepancies, a third reviewer was consulted.

Data extraction was performed independently by two authors. The extracted baseline characteristics included the first author, study region, study period, patient age, number of patients, BMI, type of MIH, and conversion. The outcomes of interest included operative time, blood loss, tumor size, overall and major postoperative complications, biliary leakage, liver failure, blood transfusion rate, mortality, and length of hospital stay. Postoperative complications were graded according to the Clavien–Dindo classification (23). Major complications were defined as Clavien–Dindo grade ≥ Ⅲ.

### Quality assessment

The quality of the included studies was assessed using the Newcastle–Ottawa Scale (NOS), which evaluates three domains: patient selection, comparability of cohorts, and assessment of outcomes (24). The total score ranges from 0 to 9. Studies with a cumulative score of at least 7 were considered high quality, those with scores of 4–6 were regarded as moderate quality, and those with scores of 0–3 were classified as low quality.

### Statistical analysis

All statistical analyses were performed using Review Manager version 5.4 software. Continuous outcomes reported as medians with ranges or medians and interquartile ranges were converted to means and standard deviations according to the method described by Hozo et al. (25). The odds ratio (OR) and weighted mean difference (WMD) were used to analyze dichotomous and continuous variables, respectively. All results were presented with 95% confidence intervals (CIs). Heterogeneity among studies was assessed using the *I*^2^statistic. *I*^2^ > 50% indicates significant heterogeneity, in which case a random-effects model was applied for the data analysis. Otherwise, a fixed-effects model was applied. Statistical significance for comparison was set at *P* < 0.05. Sensitivity analyses were conducted to explore potential sources of heterogeneity by excluding a single study at a time to evaluate its effect on the overall pooled results. Subgroup analyses were performed based on weight categories, using thresholds of BMI ≥ 25 kg/m^2^ and BMI ≥ 30 kg/m^2^.

### Publication bias

The funnel plot was used to assess the publication bias in our study.

## Results

### Study selection

**Figure 1:**
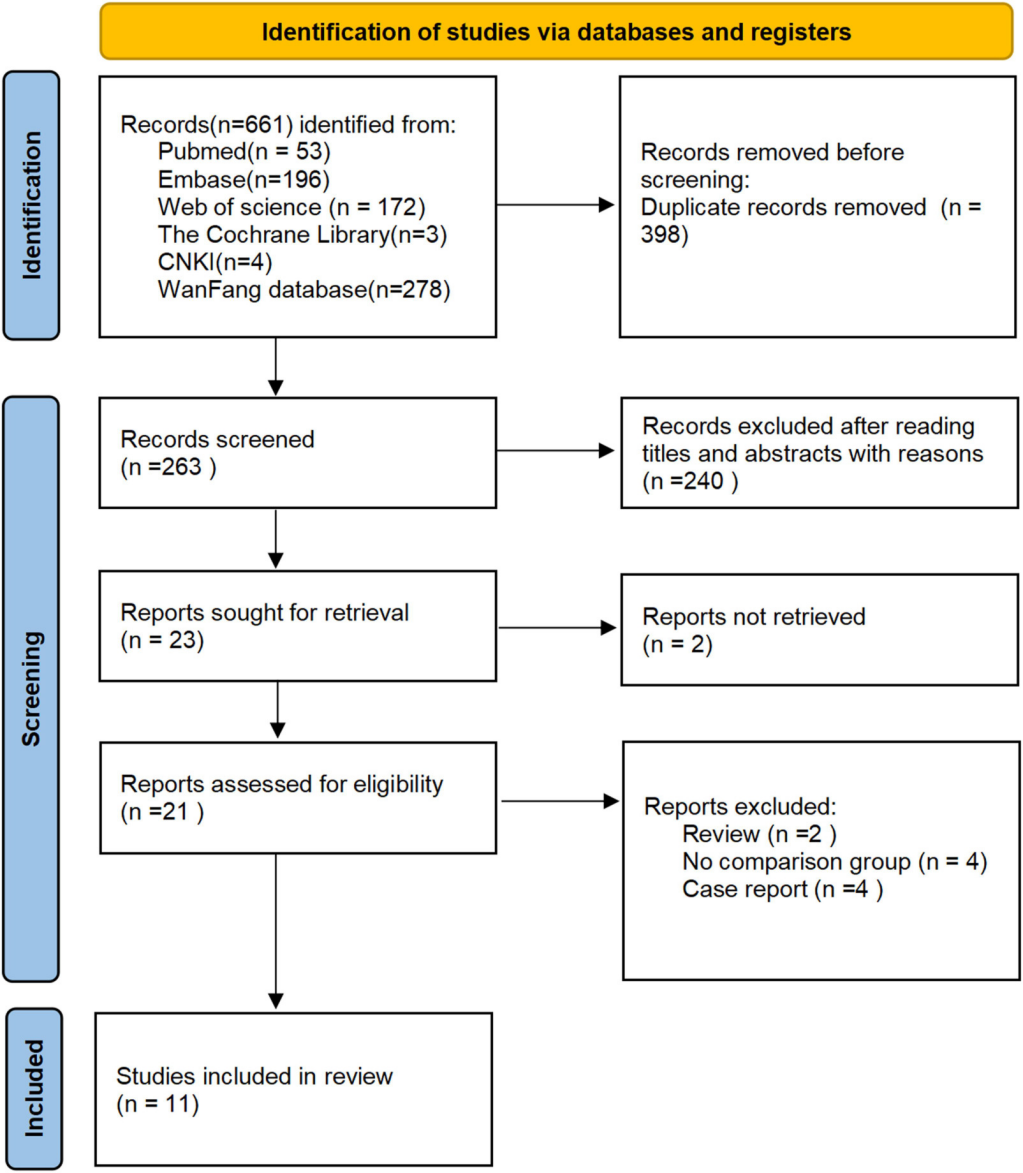
Flow diagram of the study selection process.

A total of 661 studies were identified through the initial literature search. After removing 398 duplicate records, 263 articles remained for title and abstract screening. Of these, 240 articles were excluded due to irrelevance. The full texts of the remaining 23 studies were reviewed, and 12 were excluded based on our predefined exclusion criteria. Finally, 11 retrospective studies (26–36), published between 2015 and 2023, met our inclusion criteria. The search strategies and results are presented in Figure 1.

### Characteristics and quality assessment of included studies

A total of 1,713 patients were included, with 811 patients in the MIH group and 902 patients in the OH group. The cutoff for obesity was set at a BMI of ≥25 kg/m^2^ in seven studies (26, 28, 31–35) and at ≥30 kg/m^2^ in four studies (27, 29, 30, 36). There were 678 patients in the BMI ≥ 25 kg/m^2^ subgroup and 1,035 patients in the BMI ≥ 30 kg/m^2^ subgroup. Ten studies involved laparoscopic hepatectomy (26–29, 31–36), while one study focused on robotic hepatectomy (30). In Chen's study (26), major hepatectomy was performed in 31 patients in the MIH group and 32 patients in the OH group. According to Heise's study (27), the types of hepatectomy included segmentectomy (*n* = 11, MIH: 4, OH: 7), bisegmentectomy (*n* = 20, MIH: 11, OH: 9), left hepatectomy (*n* = 3, MIH: 0, OH: 3), and right hepatectomy (*n* = 7, MIH: 5, OH: 2). In Ishihara's study (29), the procedures performed included partial resection (*n* = 26, MIH: 14, OH: 12), segmentectomy (*n* = 2, MIH: 1, OH: 1), sectionectomy (*n* = 6, MIH: 1, OH: 5), and resection of two or more sections (*n* = 4, MIH: 0, OH: 4). In Ome's study (31), the types of hepatectomy included segmentectomy (*n* = 15, MIH: 8, OH: 7), sectionectomy (*n* = 41, MIH: 14, OH: 27), bisectionectomy or hemihepatectomy (*n* = 11, MIH: 4, OH: 7), and trisectionectomy (*n* = 2, MIH: 0, OH: 2). In Yoon's study (34), the procedures included right lobectomy (*n* = 96, MIH: 48, OH: 48), left lobectomy (*n* = 95, MIH: 48, OH: 47), right anterior segmentectomy (*n* = 11, MIH: 6, OH: 5), right posterior segmentectomy (*n* = 26, MIH: 12, OH: 14), and central bisegmentectomy (*n* = 12, MIH: 6, OH: 6). In Zimmitti's study (36), 228 patients in the MIH group and 222 patients in the OH group underwent minor hepatectomy, while 225 in the MIH group and 231 patients in the OH group underwent major hepatectomy. The remaining five studies did not report the type of hepatectomy performed (28, 30, 32, 33, 35). The number of conversions ranged from 0 to 39. Ten studies were rated as high quality, while one was rated as moderate quality. The baseline characteristics of the included studies and the results of the quality assessment are presented in Table 1.

**Table 1:** Baseline characteristics of the included studies.

Study	Region	Period	Age (MIH/OH)		No. of patients		BMI		MIH type	Conversion no.	NOS score
			MIH	OH	MIH	OH	MIH	OH			
Chen et al. (26)	China	2015–2017	59.7 ± 14.7^a^	56.5 ± 12.3^a^	38	37	28.8 ± 2.8^a^	28.7 ± 2.9^a^	L	1	7
Heise et al. (27)	Germany	2015–2019	61.3 ± 10.4^a^	67.5 ± 11.0^a^	27	29	NA	NA	L	2	7
Inoue et al. (28)	Japan	2010–2018	66 (29–82)^b^	67 (45–87)^b^	34	18	26.6 (25.1–35.5)^b^	26.2 (25.1–30.8)^b^	L	1	7
Ishihara et al. (29)	Japan	2000–2019	65 (44–73)^b^	70 (44–78)^b^	16	22	33 (30.1–40.2)^b^	31.2 (30–38.1)^b^	L	1	7
Lin et al. (30)	China	2010–2020	NA	NA	21	14	NA	NA	R	NA	8
Ome et al. (31)	Japan	2010–2017	70 (41–87)^b^	67.5 (25–83)^b^	63	79	26.9 (25–33.9)^b^	27.5 (25–35.9)^b^	L	0	7
Toriguchi et al. (32)	Japan	2002–2012	64 (43–76)^b^	65 (42–88)^b^	13	69	27.4 (25.8–37.5)^b^	26.8 (25–38.2)^b^	L	0	7
Uchida et al. (33)	Japan	2010–2015	70.5 ± 9.4^a^	67.1 ± 6.2^a^	12	10	29 ± 3.7^a^	28.2 ± 2.3^a^	L	0	7
Yoon et al. (34)	Korea	2009–2018	56.38 ± 10.26^a^	55.9 ± 10.09^a^	120	120	27.14 ± 1.83^a^	27.2 ± 1.58^a^	L	NA	9
Yu et al. (35)	China	2013–2014	49.4 ± 12.7^a^	48.5 ± 8.6^a^	14	51	NA	NA	L	NA	6
Zimmitti et al. (36)	Multicountry	2009–2019	66.5 (59, 71)^c^	66 (58.1, 71.4)^c^	453	453	NA	NA	L	39	8

NA, not available; IQR, interquartile range; NOS, Newcastle–Ottawa Scale; MIH, minimally invasive hepatectomy; OH, open hepatectomy; L, laparoscope; R, robot.

^a^ Mean ± SD.

^b^ Median (range).

^c^ Median (IQR).

### Surgical outcomes

#### Operative time

**Figure 2:**
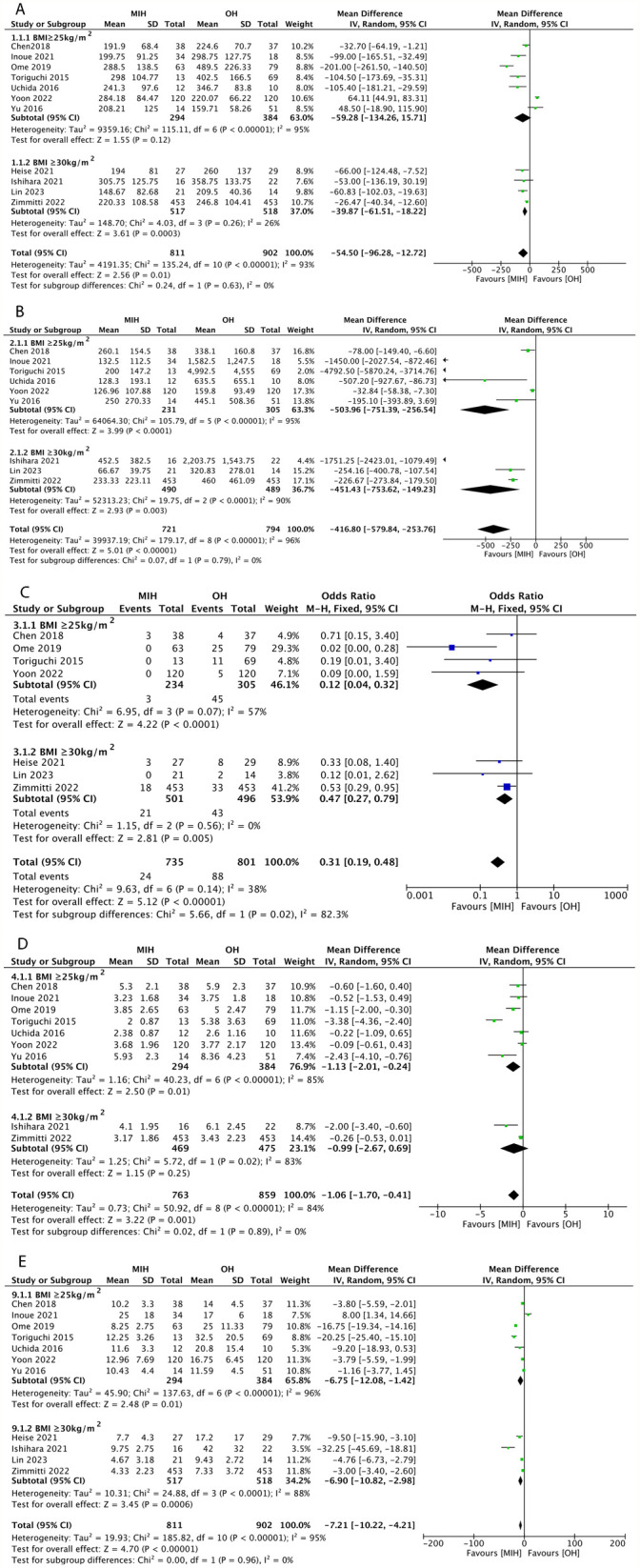
Forest plots of operative time **(A)**, blood loss **(B)**, blood transfusion **(C)**, tumor size **(D)**, and length of hospital stay **(E)**.

Operative time was reported in 11 studies (26–36). Pooled analysis using a random-effects model suggested that the operative time was shorter in the MIH group than in the OH group (1,713 patients, WMD = −54.50 min, 95% CI = −96.28 to −12.72 min, *P* = 0.01). However, a significantly high degree of heterogeneity was observed among the studies (*I*^2^ = 93%). Subgroup analysis revealed that when BMI ≥ 25 kg/m^2^, there was no significant difference in operative time between the MIH and OH groups (WMD = −59.28 min, 95% CI = −134.26 to −15.71 min, *P* = 0.12). However, when BMI ≥ 30 kg/m^2^, the operative time was shorter in the MIH group (WMD = −39.87 min, 95% CI = −61.51 to −18.22 min, *P* = 0.01) (Figure 2A).

#### Blood loss

Nine studies (26, 28–30, 32–36) reported blood loss. Pooled analysis using a random-effects model revealed that patients in the MIH group experienced less blood loss than those in the OH group (1,515 patients, WMD = −416.80 mL, 95% CI = −579.84 to −253.76 mL, *P* < 0.00001). However, high heterogeneity was observed among the studies (*I*^2^ = 96%). Subgroup analysis suggested that blood loss in the MIH group remained lower in both BMI subgroups (Figure 2B).

#### Blood transfusion rate

Seven studies reported the blood transfusion rate (26, 27, 30–32, 34, 36). Pooled analysis using a fixed-effects model showed that patients in the MIH group had a lower blood transfusion rate than those in the OH group (1,536 patients, OR = 0.31, 95% CI = 0.19–0.48, *P* < 0.00001). Heterogeneity among the studies was low (*I*^2^ = 38%). Subgroup analysis suggested that, compared with OH, the transfusion rate in the MIH group was lower in both BMI subgroups (Figure 2C).

#### Tumor size

Tumor size was reported in nine studies (26, 28, 29, 31–36). The tumor size ranged from 0.4 to 11 cm in the MIH group and 0.2 to 15 cm in the OH group. Pooled analysis using a random-effects model showed that patients with obesity in the MIH group had smaller tumors than those in the OH group (1,622 patients, WMD = −1.06 cm, 95% CI = −1.70 to −0.41 cm, *P* = 0.001). However, a high degree of heterogeneity was observed among the studies (*I*^2^ = 84%). When BMI ≥ 25 kg/m^2^, the tumor size ranged from 0.4 to 11 cm in the MIH group and 0.2 to 15 cm in the OH group. Pooled analysis suggested that tumor size was smaller in the MIH group than in the OH group (WMD = −1.13 cm, 95% CI = −2.01 to −0.24 cm, *P* = 0.01). However, when BMI ≥ 30 kg/m^2^, the tumor size ranged from 1.7 to 9.5 cm in the MIH group and 2 to 12 cm in the OH group. Subgroup analysis revealed no significant difference in tumor size between the MIH and OH groups (WMD = −0.99 cm, 95% CI = −2.67 to 0.69 cm, *P* = 0.25) (Figure 2D).

#### Length of hospital stay

The length of hospital stay was reported in 11 studies (26–36). Pooled analysis using a random-effects model showed that patients with obesity in the MIH group had a shorter postoperative hospital stay than those in the OH group (1,713 patients, WMD = −7.21 days, 95% CI = −10.22 to −4.21 days, *P* < 0.00001). In addition, a high degree of heterogeneity was observed among the studies (*I*^2^ = 95%). Subgroup analysis suggested that the hospital stay in the MIH group was shorter in both BMI subgroups (Figure 2E).

#### Overall complications

**Figure 3:**
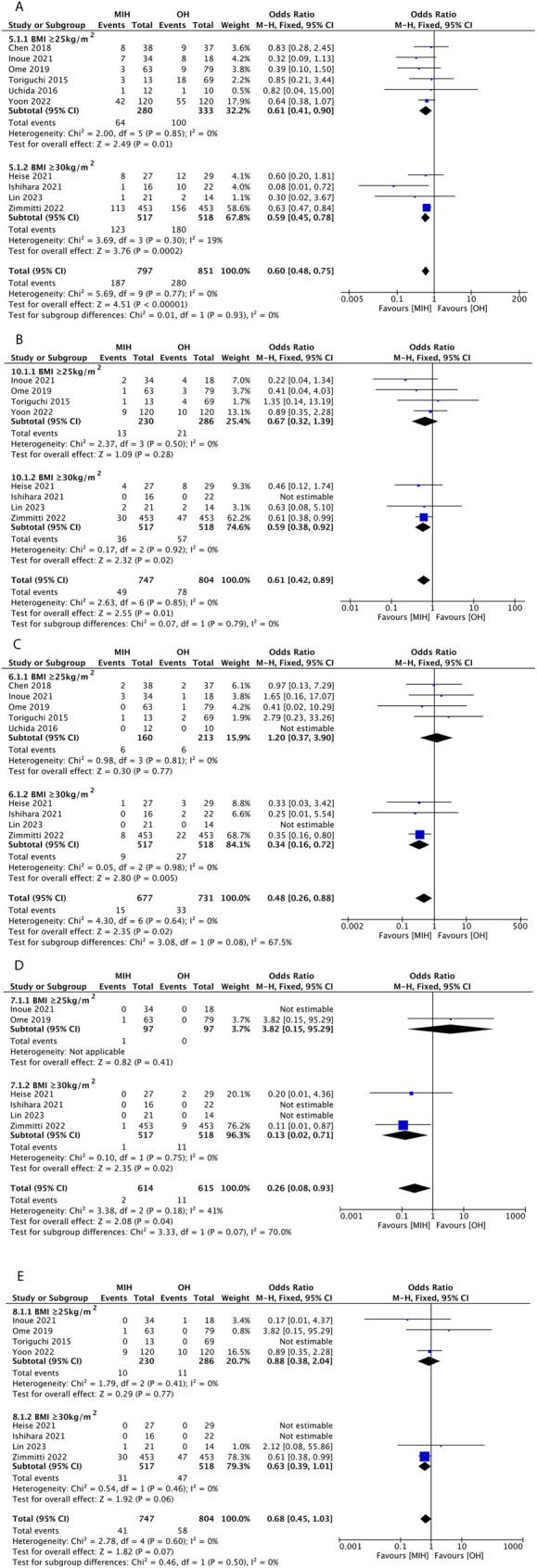
Forest plots of overall complications **(A)**, major complications **(B)**, biliary leakage **(C)**, liver failure **(D)**, and mortality **(E)**.

Ten studies reported overall postoperative complications (26–34, 36). Pooled analysis using a fixed-effects model showed that the incidence of overall postoperative complications was lower in the MIH group (1,648 patients, OR =  0.60, 95% CI = 0.48–0.75, *P* < 0.0001). Heterogeneity among the studies was low (*I*^2^ = 0%). Subgroup analysis suggested that the rate of overall complications in the MIH group remained lower in both BMI subgroups (Figure 3A).

#### Major complications

Eight studies reported major postoperative complications (27–32, 34, 36). Pooled analysis using a fixed-effects model revealed that the incidence of major postoperative complications was lower in the MIH group (1,551 patients, OR =  0.61, 95% CI = 0.42–0.89, *P* = 0.01). No significant heterogeneity was observed among the studies (*I*^2^ = 0%). Subgroup analysis revealed that when BMI ≥ 25 kg/m^2^, there was no significant difference in major complications between the MIH and OH groups (OR =  0.67, 95% CI = 0.32–1.39, *P* = 0.28). However, when BMI ≥ 30 kg/m^2^, the incidence of major complications was lower in the MIH group (OR = 0.59, 95% CI = 0.38–0.92, *P* = 0.02) (Figure 3B).

#### Biliary leakage

Biliary leakage was reported in nine studies (26–33, 36). Pooled analysis using a fixed-effects model suggested that the occurrence of biliary leakage was lower in the MIH group (1,408 patients, OR =  0.48, 95% CI = 0.26–0.88, *P* = 0.02). There was no significant heterogeneity among the studies (*I*^2^ = 0%). Subgroup analysis revealed that when BMI ≥ 25 kg/m^2^, there was no significant difference in biliary leakage between the MIH and OH groups (OR =  1.20, 95% CI = 0.37–3.90, *P* = 0.77). However, when BMI ≥ 30 kg/m^2^, the biliary leakage was lower in the MIH group (OR = 0.34, 95% CI = 0.16–0.72, *P* = 0.005) (Figure 3C).

### Liver failure

Liver failure was reported in three studies (27, 31, 36). Pooled analysis using a fixed-effects model suggested that the occurrence of liver failure was lower in the MIH group (1,104 patients, OR =  0.26, 95% CI = 0.08–0.93, *P* = 0.04). Low heterogeneity was observed among the studies (*I*^2^ = 41%). Subgroup analysis revealed that when BMI ≥ 25 kg/m^2^, there was no significant difference in liver failure between the MIH and OH groups (OR =  3.82, 95% CI = 0.15–95.29, *P* = 0.41). However, when BMI ≥ 30 kg/m^2^, liver failure was lower in the MIH group (OR = 0.13, 95% CI = 0.02–0.71, *P* = 0.02) (Figure 3D).

### Mortality

Mortality was reported in eight studies (27–32, 34, 36). Pooled analysis using a fixed-effects model suggested that there was no significant difference in mortality between the MIH and OH groups (1,551 patients, OR = 0.68, 95% CI = 0.45–1.03, *P* = 0.07). There was no significant heterogeneity among the studies (*I*^2^ = 0%). Subgroup analysis suggested no significant difference in mortality between the MIH and OH groups in either subgroup (Figure 3E).

### Sensitivity and subgroup analyses

Sensitivity analysis was conducted by excluding a single study at a time to evaluate the effect on overall pooled results. However, the heterogeneity did not change significantly.

Due to differences in the global definition of obesity (37, 38), we performed subgroup analyses using BMI thresholds of ≥25 and ≥30 kg/m^2^. The subgroup analysis showed that in the BMI ≥ 25 kg/m^2^ subgroup, no significant differences were found between the MIH and OH groups in operative time, major complications, biliary leakage, or liver failure. However, in the BMI ≥ 30 kg/m^2^ subgroup, MIH was associated with a shorter operative time and lower rates of major complications, biliary leakage, and liver failure.

### Publication bias

**Figure 4:**
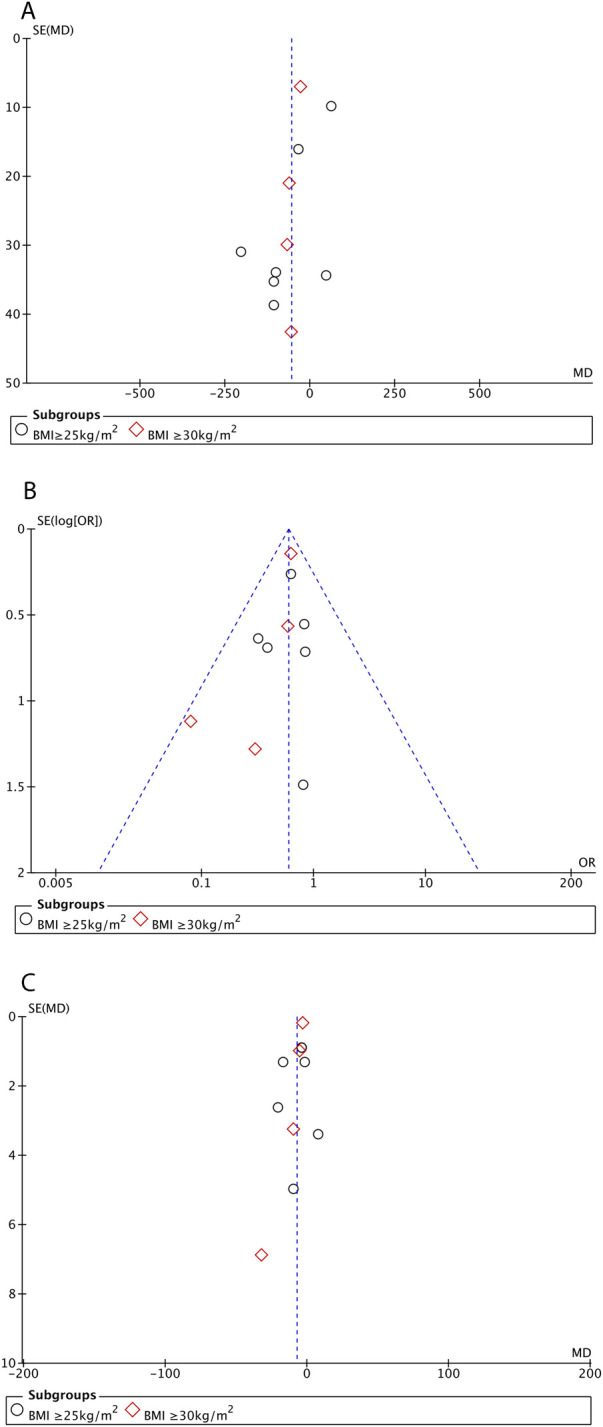
Funnel plot for operative time **(A)**, overall postoperative complications **(B)**, and length of hospital stay **(C)**.

Publication bias was evaluated using funnel plots for operative time (Figure 4A), overall complications (Figure 4B), and length of hospital stay (Figure 4C). The distribution of scatter spots was symmetrical on both sides of the funnel plots, suggesting no evidence of publication bias.

## Discussion

This is the first meta-analysis to compare the surgical outcomes of MIH vs. OH in patients with obesity, including 11 studies with a total of 1,713 patients. The results indicated that patients in the MIH group experienced shorter operative times, reduced blood loss, lower blood transfusion rates, fewer postoperative complications, and shorter hospital stays. However, no significant difference in mortality was observed between the MIH and OH groups. MIH proves to be a safe and effective treatment option for patients with obesity.

Previous studies comparing patients with and without obesity have indicated that patients with obesity required longer operative times during traditional OH (39, 40). However, with advancements in minimally invasive techniques, MIH has demonstrated advantages in liver surgery, achieving operative times comparable to those of OH (41). In our study, patients with obesity in the MIH group had shorter operative times than those in the OH group. This finding may be attributed to the magnified surgical view, smaller incisions, and the enhanced stability and flexibility of the robotic platform used in MIH (15, 16). While Berardi and Truant reported that obesity increased conversion rates and prolonged operative times in MIH (18, 40), our findings differ. This discrepancy may be explained by the fact that their studies primarily focused on major hepatectomy, a context in which MIH tends to require longer operative times than OH (42, 43). The studies included in our analysis exhibited high heterogeneity in operative time. Although sensitivity analysis was performed, the source of this heterogeneity remained unclear. It may stem from differences in surgical expertise, the stage of the surgeons’ learning curve, and variations in hospital volume. First, varying levels of surgical expertise may lead to significant differences in operative time. Second, the learning curve for complex procedures like MIH is substantial, and consequently, operative time may vary depending on the surgeons’ stage along the learning curve. Third, surgeons in high-volume centers are likely to have more experience and may have already surpassed the learning curve compared with those in lower-volume centers. All these factors may contribute to the high heterogeneity in operative time.

Obesity is an independent risk factor for increased perioperative blood loss in patients undergoing hepatectomy (18, 40, 44). Our study found that, compared with OH, MIH was associated with less blood loss and a lower blood transfusion rate. This finding is consistent with outcomes observed in patients with obesity undergoing laparoscopic gastrectomy (45). These benefits may be attributed to several advantages of MIH. First, the effect of pneumoperitoneum in MIH is more pronounced, which may reduce the impact of obesity and lead to less bleeding compared with OH (44). Second, the high clarity and magnification provided by MIH allow surgeons to better identify intrahepatic blood vessels. Third, advancements in MIH equipment, particularly in controlling bleeding, play a significant role in reducing blood loss and the need for transfusion. In contrast, OH lacks these advantages during surgery. In addition, excess adipose tissue in patients with obesity can place the liver deeper within the abdominal cavity, limiting the operative field visibility during OH and potentially increasing bleeding.

As one of the attendant complications of obesity, steatotic liver is particularly vulnerable to ischemia–reperfusion injury, which increases the risk of postoperative morbidity and mortality following liver resection (46). In addition, surgical outcomes have demonstrated an association between obesity and increased surgical complications (39, 47). Evidence from studies of hepatectomy suggests that patients with obesity re more likely to experience hepatic-specific perioperative complications (48). However, compared with traditional OH, these risks were not observed in patients with obesity undergoing MIH (49). Our study also revealed a lower occurrence of hepatic-specific complications, such as biliary leakage and liver failure, in the MIH group. Furthermore, both overall and major complications were less frequent in the MIH group. These findings may be attributed to several advantages of MIH. First, MIH requires smaller incisions compared with OH. Previous studies have reported significantly higher rates of abdominal wall complications in patients with obesity undergoing OH (47). Second, the lower blood transfusion rate observed in MIH may contribute to reduced postoperative complications, as blood transfusion has been associated with adverse effects on the occurrence of complications (50). Third, reduced manipulation and compression of the liver during MIH may minimize liver injury. Fourth, decreased inflammatory and physiological stress response associated with MIH may also help reduce postoperative complications (51). While Berardi et al. reported an increase in postoperative complications with increasing BMI among patients undergoing MIH (18), we believe that this finding may be partly explained by the fact that their study focused on major hepatectomy, as previous studies have shown that longer operative times and major hepatectomy are associated with an increased risk of postoperative complications in patients with obesity (52, 53). In addition, no significant difference in mortality was observed between the MIH and OH groups in our study, which aligns with findings from previous research (52).

Literature has shown that patients with obesity undergoing OH tend to have longer hospital stays compared with patients of normal weight (48). However, in minimally invasive methods, our study demonstrated that patients in the MIH group were discharged earlier than those in the OH group. As reported in many previous studies, a shorter hospital stay is one of the advantages of MIH (41, 43, 44). This may be attributed to the reduced invasiveness, less pain, earlier recovery of gastrointestinal function, and fewer postoperative complications associated with MIH (41). However, the high heterogeneity among studies may be influenced by factors such as surgeons’ technical proficiency, hospital volume, and variations in discharge criteria across hospitals.

A high BMI is associated with an increased risk of cancer (7). Studies have shown that obesity creates a supportive environment for tumor cell proliferation, with potential mechanisms including insulin resistance, endogenous hormone production, adipokine secretion, angiogenesis, obesity-induced hypoxia, immune modulation, and inflammatory cytokines (54). While obesity has been linked to reduced overall survival, recent studies have reported conflicting results regarding prognosis in patients with obesity (55, 56). Therefore, the relationship between obesity and long-term prognosis remains controversial. Petrelli et al. reported that obesity was a risk factor for overall survival in patients with breast cancer but a protective factor for patients with gastrointestinal and lung cancers compared with those without obesity (55, 57, 58). Similar findings have also been observed in studies of renal cancer (59, 60). However, in colorectal cancer, no significant difference in cancer-specific survival has been found between patients with obesity and those of normal weight (61). For liver cancer, the literature also presents contradictory results. While obesity is considered an independent risk factor for the development of liver cancer and has been associated with poor prognosis after liver resection (20, 62), some studies have found no adverse effects of obesity on surgical outcomes and prognosis (21, 63). In our study, only one included study reported long-term outcomes, and no significant difference was found between MIH and OH (34). Therefore, long-term prognosis was not analyzed in our meta-analysis due to the limited available data. Most current studies involving patients with obesity and liver cancer undergoing MIH focus on perioperative outcomes, with insufficient data on long-term prognosis. Further research is therefore needed to investigate the long-term outcomes of these patients after MIH.

Several limitations of our study should be acknowledged. First, BMI classifications for obesity vary among countries (37, 38), leading to non-uniform definitions of obesity among the included studies. This inconsistency may affect the rigor of our conclusions. Second, the pooled results for some variables exhibited high heterogeneity, which may be attributed to differences in surgeons’ learning curve, study designs, and levels of surgical expertise. Third, tumor size was smaller in the MIH group than in the OH group, suggesting the presence of selection bias. Finally, all studies included in our meta-analysis were retrospective, which may also introduce a degree of selection bias.

## Conclusion

This study demonstrates that, compared with OH, MIH offers several advantages for patients with obesity, including shorter operative time, fewer complications, reduced blood loss, lower transfusion rates, and shorter hospital stays. Therefore, MIH represents a safe and feasible approach for liver resection in patients with obesity. However, given the retrospective nature of the included studies, these advantages of MIH should be further confirmed through prospective randomized controlled trials.

## Data Availability

The original contributions presented in the study are included in the article/Supplementary Material, further inquiries can be directed to the corresponding author.
