# Supplementary material for: Minimally invasive vs. open hepatectomy in patients with obesity and liver tumors: a systematic review and meta-analysis
Source: Front Surg. 2026 Apr 15;13:1718991. doi: 10.3389/fsurg.2026.1718991 (PMC13126306; doi:10.3389/fsurg.2026.1718991)
Supplement: Supplementary file 1 [file Table1.doc]

**The Newcastle-Ottawa Scale (NOS)**

| Study | Selection | | | | Comparability | Outcome | | | Score |
| --- | --- | --- | --- | --- | --- | --- | --- | --- | --- |
|  | Representativeness of exposed cohort | Selection of non-exposed cohort | Exposure | Outcome of interest not present at start |  | Assessment of outcome | Follow-up long enough | Adequacy of follow- up |  |
| Chen 2018 (26) | 1 | 1 | 1 | 1 | 1 | 1 | 1 | 0 | 7 |
| Heise 2021 (27) | 1 | 1 | 1 | 1 | 1 | 1 | 1 | 0 | 7 |
| Inoue2020 (28) | 1 | 1 | 1 | 1 | 1 | 1 | 1 | 0 | 7 |
| Ishihara2021 (29) | 1 | 1 | 1 | 1 | 1 | 1 | 1 | 0 | 7 |
| Lin2023 (30) | 1 | 1 | 1 | 1 | 2 | 1 | 1 | 0 | 8 |
| Ome2019 (31) | 1 | 1 | 1 | 1 | 1 | 1 | 1 | 0 | 7 |
| Toriguchi2015 (32) | 1 | 1 | 1 | 1 | 1 | 1 | 1 | 0 | 7 |
| Uchida2016 (33) | 1 | 1 | 1 | 1 | 1 | 1 | 1 | 0 | 7 |
| Yoon2022 (34) | 1 | 1 | 1 | 1 | 2 | 1 | 1 | 1 | 9 |
| Yu2016 (35) | 1 | 1 | 1 | 1 | 1 | 1 | 0 | 0 | 6 |
| Zimmitti2022 (36) | 1 | 1 | 1 | 1 | 2 | 1 | 1 | 0 | 8 |
